# Supplementary material for: Are carbon emissions trading and green financial instruments synergistic? -Comprehensive quantitative research based on content analysis
Source: PLoS One. 2024 Mar 7;19(3):e0298601. doi: 10.1371/journal.pone.0298601 (PMC10919601; doi:10.1371/journal.pone.0298601)
Supplement: S1 Data — (ZIP) [file pone.0298601.s001.zip › Data/地级市Z_F工作报告.zip!/░▓╗╒/╨√│╟╩╨/└┤╫╘╣1⁄2╓┌║┼í╛┬φ┐╦╩2╛▌═°í┐_2003.docx]

各位代表：

　　现在，我代表市人民政府向大会作政府工作报告，请予审议，并请市政协委员和其他列席会议的同志提出意见。

　 　 　 　 　 　　　 　 　 一、2002年工作回顾

　　刚刚过去的2002年，在省委省政府和市委的领导下，全市人民坚持以邓小平理论和“三个代表”重要思想为指导，认真学习贯彻江泽民同志“5．31”重要讲话和党的十六大精神，努力适应加入世贸组织的新形势，以加速工业化为核心，以招商引资为主要抓手，大力整治和改善经济发展环境，加快结构调整，深化各项改革，扩大对外开放，扎实推进宣城经济融入苏浙沪经济圈的历史进程，整体经济呈现出增速加快、质量提高、亮点增多、后劲增强的良好发展态势，各项社会事业同步推进。

　　整体经济增速加快，结构调整出现积极变化。预计全市国内生产总值可达181亿元，比上年增长7.4%。完成财政总收入11.08亿元，按可比口径增长8.1%，其中地方财政收入6.5亿元，按可比口径增长9.3%。城镇居民人均可支配收入5873元，增长7%。农民人均纯收入2400元，增长3 %。工业经济在调整中保持较快增长，质量和效益继续改善。规模以上工业增加值比上年增长7.5%，工业固定资产投资增长105%，工业用电量、实现利税和经济效益综合指数均比上年有较大幅度的增长。一批优势企业通过资产重组和技术创新，支撑作用更加明显。一批中小企业特别是困难企业，通过招商引资和实施民营化改组改造，整体活力有所增强。农村经济稳步发展，农业产业化经营全面推进。预计全年粮食总产可达110万吨，农业增加值比上年增长4.2%。优质粮油、高效经济作物和畜牧水产业、经济林果业发展迅速，森林生态网络体系建设全面启动。农产品质量明显提高，无公害绿色食品达到34个，比上年增加一倍，规模化养禽继续保持全省领先水平。农业信息服务取得新的进展，率先在全省县县开通农技110服务热线。农村劳动力转移步伐加快，非农产业收入进一步提高。第三产业发展势头较好，占国内生产总值的比重比上年提高0.2个百分点。金融保险业稳健运行，创建金融安全区工作取得积极成效，年末金融机构各项存款余额126.01亿元，比年初增加18.28亿元；各项贷款余额111.46亿元，比年初增加17.62亿元。旅游业蓬勃发展，旅游总收入达8.5亿元，增长29%。房地产业方兴未艾，首届宣城房交会成交额达3.9亿元。非公有制经济发展加快，全市个体工商户已发展到7.6万户，私营企业达到3480家，个体私营经济占全市经济总量的份额超过30%。

　　各项改革稳步推进，对外开放进一步扩大。企业改革继续向纵深拓展，改制面和劳动关系转换面分别达到90%和85%。市直企业改革特别是驻芜企业改革取得新的进展，禽蛋厂和皖南建筑总公司先后实施关闭破产。市国诚投资有限公司、国诚典当公司和市中小企业信用担保公司挂牌运营。农村税费改革继续深化，新的农村税费制度框架基本确立。社会保障工作得到加强，国有企业下岗职工基本生活费和离退休人员基本养老金做到按时足额发放。下岗职工出中心工作稳步推进，市场导向的就业机制初步形成。城镇居民最低生活保障覆盖面逐步扩大，“应保尽保”目标基本实现。公共财政支出改革全面实施，财政运行质量良好。市县两级党政机构改革工作顺利完成，全市党政群机关和直属事业单位机构由改革前的513个减少到404个，部门内设机构由改革前的1867个减少到1632个，人员编制减少近20%，机关建设和工作作风出现了新的气象。对外贸易增势强劲，全市外贸进出口总额预计可突破8000万美元，比上年增长30%以上，其中出口5750万美元，增长39.2%，占省下达任务的133.7 %，增幅位居全省前列。招商引资势头良好，全年共新批外商投资项目16个，45个项目有外资到位，实际利用外资2587.6万美元，占省下达任务的129.4%。引进市外投资项目527个，总投资41.15亿元，已到位资金14.79亿元，其中省外资金11.33亿元，来自苏浙沪地区的投资超过90%。成功举办了第二届宣城旅游招商暨促销洽谈会、外商投资暨农业利用外资洽谈会和宁国山核桃节。宣城经济技术开发区和各类工业园区建设取得新的进展，落户企业不断增多，投资规模进一步扩大。新鸿发、飞鹰、新和成皖南药业等一批新引进的大项目，相继开工兴建。

　　基础设施建设步伐加快，整治和改善经济发展环境工作取得阶段性成果。全年固定资产投资预计可达44.4亿元，比上年增长21%。农村电网改造二期工程、移民建镇二期工程、市中心血站、泾县国家粮库等国债项目按计划基本完成，广德220kv输变电工程等项目相继建成并投入运营。马山埠闸、白沙水库、郎溪县城区防洪工程等重点建设项目进展顺利，双桥闸开工兴建。交通“九路八桥”工程启动实施，芜宣高速公路宣城段、205国道泾南段和郎溪新老郎川河复桥等相继竣工，318国道广祠段高速公路、104省道河唐线改造等正在抓紧建设之中。城市建设力度加大，环城大道开发区中段、梅溪路开发区段改造、火车站游园广场和宣湖路等重点市政工程项目如期完成。土地市场进一步规范，城市经营性用地公开招标拍卖工作取得积极成效。市区部分道路和地段得到有效整治，市容市貌有了新的改观。集中开展了整治和改善经济发展环境工作，深入查摆问题，认真组织整改，大力推进行政审批制度、行政收费制度和检查（评比、督查）办法改革，取消和调整行政审批211项，清理不合理收费129项。全面推广“一站式”高效服务方式、政务公开和办文办事限时制度，市政务服务中心的服务项目已达到233项，全年办结各类业务5.2万件，行政收费5921万元。广泛开展政风评议和创建“人民满意的基层站所”活动，政府部门的行政行为进一步规范，服务意识不断增强。

　　科技教育和各项社会事业取得新的发展，精神文明和民主法制建设得到加强。先进适用技术推广力度不断加大，科技对经济增长的贡献率进一步提高。基础教育得到加强，素质教育全面推开，学前教育、职业教育、民办教育和普通高等教育有了新的发展。全市有6所学校进入省级示范高中行列，高中阶段招生规模继续扩大，宣城职业技术学院获省政府正式批准。中小学布局调整和危房改造工作成效显著，改造危房16.3万平方米。人口与计划生育综合改革扎实推进，人口出生率和自然增长率分别控制在8‰和3.5‰以内。艺术创作、群众性文化活动更加丰富，文化市场管理逐步规范。全民健身运动广泛开展，竞技体育在省“十运会”上取得较好成绩。城乡卫生条件逐步改善，医疗卫生体制和药品流通体制改革积极推进。广电城域网改造升级和数字电视项目启动实施，广播电视节目质量不断提高，“关注”、“百姓热线”等栏目受到群众欢迎。扶贫开发工作取得新的进展，贫困地区的群众生活逐步改善。对外交流继续扩大，市政府代表团成功访问日本川之江市和川之江市体育交流代表团访问宣城，使两市之间的交流与合作得到加强。公民道德教育和精神文明创建活动深入开展，人民群众的思想道德素质和文明程度有了新的提高。“四五”法制宣传教育有效推进。第五届村委会统一换届工作顺利完成，社区建设得到加强。市政府全年共办理人大议案1件、代表建议61件，政协委员提案59件，办理质量不断提高。审计、统计、气象、地震、民族、宗教、外事、侨务、档案等部门积极服务经济建设，工商、税务、物价、环保、质量技术监督、药品监督管理等部门依法行政，做出了新的成绩。社会治安综合治理力度加大，人民来信来访工作进一步改进，群体性事件和集体上访得到及时稳妥处理。政风建设和反腐倡廉工作深入开展，公款吃喝现象有所遏制。国防后备力量建设和“双拥”工作得到加强。

　　各位代表，过去的一年，我们不仅较好完成了全市经济和社会发展的各项任务，更为重要的是，通过认真学习贯彻党的十六大精神，推进宣城经济融入苏浙沪经济圈的发展战略，极大地激发了全市人民加快发展的紧迫感和责任感，广大干部群众的思想观念和精神面貌正在发生深刻变化，经济和社会生活正在焕发出新的生机和活力，想发展、议发展、谋发展已经成为全市上下的最强音。这些成绩是省委省政府和市委正确领导的结果，是全市上下团结奋斗、扎实工作的结果。在此，我谨代表市人民政府，向为宣城改革和发展付出辛勤劳动的全市广大干部群众，向给予市政府工作大力支持与有效监督的人大代表、政协委员和各民主党派、工商联、无党派人士，向为宣城各方面工作作出积极贡献的人民解放军指战员、武警官兵、政法干警和中央、省属各单位，向所有关心、支持宣城改革开放和现代化建设的同志们、朋友们表示崇高的敬意和衷心的感谢！

　　在肯定成绩的同时，我们也清醒地看到，当前我市经济社会生活中还存在不少困难和问题。主要表现在：经济总量不大，产业结构层次较低，工业化和城市化水平不高，整体经济的竞争力还不强；农村非农产业发展不快，农民增收的难度加大；社会保障体系还不够完善，就业和再就业矛盾比较突出，部分群众生活比较困难；政府职能转变还不够到位，整治和改善经济发展环境工作有待进一步深化和拓展等。对这些问题，我们将采取有效措施，认真加以解决.
